# Synergistic Antipseudomonal Effects of Synthetic Peptide AMP38 and Carbapenems

**DOI:** 10.3390/molecules21091223

**Published:** 2016-09-12

**Authors:** Héctor Rudilla, Ester Fusté, Yolanda Cajal, Francesc Rabanal, Teresa Vinuesa, Miguel Viñas

**Affiliations:** 1 Molecular Microbiology and Antibiotics, Department of Pathology and Experimental Therapeutics, Medical School-IDIBELL, University of Barcelona, Hospitalet, Barcelona 08907, Spain; hectorrudilla@gmail.com (H.R.); esterfustedominguez@ub.edu (E.F.); tvinuesa@ub.edu (T.V.); 2 Department of Public Health, Mental Health and Perinatal Nursing, US of Nursing, University of Barcelona, Hospitalet, Barcelona 08907, Spain; 3 Department of Physical Chemistry and Institute of Nanoscience and Nanotechnology, University of Barcelona, Barcelona 08028, Spain; ycajal@ub.edu; 4 Department of Organic Chemistry, University of Barcelona, Barcelona 08028, Spain; frabanal@ub.edu; 5 Cooperativa De Ensino Superior Politécnico Universitário, Instituto de Investigação e Formação Avançada em Ciências e Tecnologias da Saúde (CESPU, IINFACTS), Gandra 4585-116, Portugal

**Keywords:** *Pseudomonas*, antimicrobial peptides, synergism, biofilm eradication

## Abstract

The aim was to explore the antimicrobial activity of a synthetic peptide (AMP38) and its synergy with imipenem against imipenem-resistant *Pseudomonas aeruginosa*. The main mechanism of imipenem resistance is the loss or alteration of protein OprD. Time-kill and minimal biofilm eradication concentration (MBEC) determinations were carried out by using clinical imipenem-resistant strains. AMP38 was markedly synergistic with imipenem when determined in imipenem-resistant *P. aeruginosa*. MBEC obtained for the combination of AMP38 and imipenem was of 62.5 μg/mL, whereas the MBEC of each antimicrobial separately was 500 μg/mL. AMP38 should be regarded as a promising antimicrobial to fight MDR *P. aeruginosa* infections. Moreover, killing effect and antibiofilm activity of AMP38 plus imipenem was much higher than that of colistin plus imipenem.

## 1. Introduction

*Pseudomonas aeruginosa* is a nosocomial opportunistic pathogen causing a wide variety of both acute and chronic infections, such as pneumonia, bacteraemia, and urinary tract infections. Immunocompromised patients and those suffering cystic fibrosis show a particularly high susceptibility to infection by this microorganism [1].

Moreover, the increasing frequency of the isolation of multidrug-resistant *Pseudomonas aeruginosa* (MDRPA) is a major cause for concern. Antimicrobial resistance in *P. aeruginosa* is caused by three well-known basic mechanisms: uptake and efflux balance, target modifications, and inactivation of the drug [2,3].

Carbapenems—particularly imipenem—are broad-spectrum antimicrobials commonly used for the treatment of MDRPA infections. Their mechanism of action is based in the inhibition of the third step of the synthesis of bacterial cell wall by binding to certain penicillin-binding proteins (PBPs). Imipenem is active against a wide variety of microorganisms, both Gram-negative (*P.* *aeruginosa*, *Acinetobacter* spp.) and Gram-positive (*Streptococcus pneumoniae* and *Enterococcus faecalis*) [4,5]. The use of this antimicrobial is in principle restricted to severe infections where microorganisms have acquired resistance to other drugs. Unfortunately, imipenem-resistant isolates have emerged in the last few years. The main mechanisms of resistance to carbapenems in *P. aeruginosa* are the loss or the alteration of the OprD porin, an outer membrane protein that facilitates the diffusion of basic amino acids and allows penetration of carbapenems (and particularly imipenem) into the bacterium, the production of β-lactamases, and the overexpression of efflux pumps [6,7]. The loss of porin OprD and the overproduction of extended-spectrum cephalosporinases (ESACs) that weakly hydrolyze carbapenems has been observed in 100% and 92% of the meropenem-resistant isolates, respectively. *P. aeruginosa* can very often accumulate different resistance mechanisms, including ESAC production, leading to carbapenem resistance [8]. The emergence of multidrug-resistant Gram-negative bacteria, as well as the lack of new drugs to combat them, has stimulated the rescue of polymyxins as therapeutic options. Polymyxins are cyclic peptides with antimicrobial action that have been available since 1949, although they were left largely unused during the seventies because of their nephrotoxicity and the availability of less toxic antimicrobials to which bacteria had not yet developed resistance [9]. 

The most known polymyxin is colistin; like other cationic polypeptides, colistin is an amphipathic compound. It is believed that this amphipathic nature is relevant to its activity against bacteria. The hydrophilic part (positively charged) would interact with the negatively-charged bacterial outer membrane. In this way, the hydrophobic part of the polypeptide would be allowed entry through the bacterial cytoplasmic membrane. Another mechanism proposed is the interaction with binding sites of divalent cations such as Mg^2+^ and Ca^2+^; this competitive binding would disturb the properties and stability of the outer membrane. Ultimately, bacterial death would be due to the insertion of the peptide into the cytoplasmic membrane, forming channels where small molecules, ions, and even proteins would pass—and that could also eventually be used as a way for other antimicrobial agents to penetrate the bacteria [10,11]. 

The exploration of antimicrobial peptides (AMP) mimicking the structure and mechanism of action of polymyxins can be regarded as a main goal of this field of research. Such synthetic AMPs would allow opening perspectives in order to reduce secondary effects and enhance antimicrobial action. Moreover, there are several experimental and clinical studies regarding the synergistic activity of colistin with other antimicrobial agents against MDRPA (azlocillin, aztreonam, ceftazidime, or ciprofloxacin, among others) [12,13,14]. Carbapenems are the most commonly used antibiotics to test these eventual synergies, although there is a huge heterogeneity in the published results [15]. The efficacy of colistin in monotherapy against beta-lactam-susceptible bacteria is lower than that of β-lactams, but when used combined with other antimicrobials, they reach higher effectiveness [16].

The increase in the use of polymyxins has resulted in the emergence of worldwide polymyxin-resistant *P. aeruginosa* isolates. This resistance lies behind a reduced negative charge on the bacterial outer membrane which has been shown to be a specific modification of lipid A in the LPS [17]. The synthesis of safe and effective antimicrobial peptides in the laboratory could open new frontiers to combating multidrug resistance—an unlimited number of new molecules with antimicrobial activity could be designed. The challenge lies in finding new molecules with higher activity and lower toxicity than conventional known drugs.

The aim of this study was to explore the antimicrobial activity of a novel synthetic cyclolipopeptide analog of polymyxin (AMP38) and its synergy with carbapenems in order to contribute to the improvement of treatments for infections caused by carbapenem-resistant *P. aeruginosa*. AMP38 was previously described as one of the most effective peptides of a series synthesized by Rabanal et al. [18].

## 2. Results

**Figure 1:**
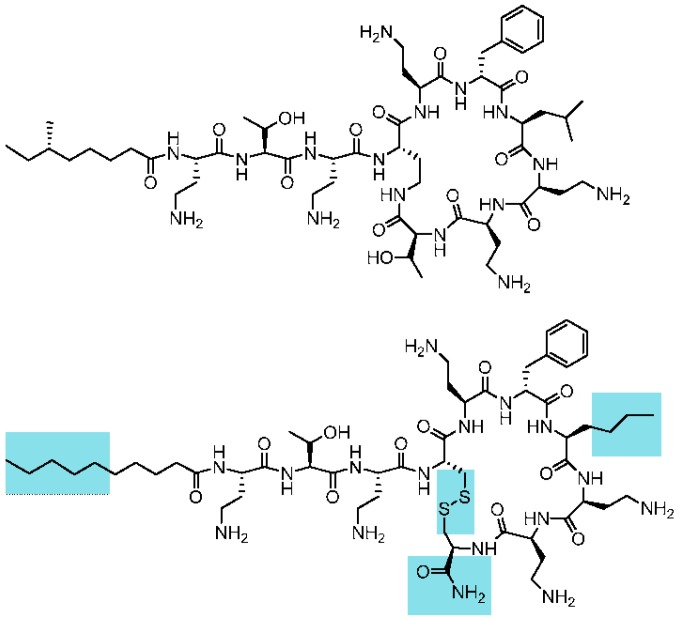
Chemical structure of the cyclolipopeptide analogue of polymyxin AMP38 (**below**) and of natural polymyxin B (**above**). The structural and chemical features modified in the analogue with respect to polymyxin are highlighted in blue.

Clinically used polymyxins are cyclic lipopeptides with a tail-to-side-chain amide bond (Figure 1). The fatty acid tail typically contains a stereocenter. Although their synthesis is accessible using chemical methods, a first goal was to reduce the complexity of the macrocyclic backbone scaffold to facilitate the generation of analogues and future preparation at high scale. A plausible approach would consist of substituting the amide bond by an isosteric and more chemically-accessible link, such as a disulfide bond. Disulfides are also chemical linkages present in some cyclic peptide drugs, as discussed below. Loop structures linked by disulfide bonds are not uncommon in cyclic AMPs. Examples are found in bactenicin (from cattle), lactoferricin, brevinins (from frog). The similarity between the macrocyclic heptapeptide structure in ranalexin and polymyxin was reported by Zasloff [19]. Similarly, Porro et al. [20] described all-l-amino acid polymyxin-derived cyclic heptapeptides capable of binding to lipid A having the capability to detoxify bacterial endotoxin (LPS) in vitro, but lacking antimicrobial activity. Altogether, this background and these structural data supported the theoretical feasibility of substituting the tail-to-side-chain amide bond with a disulfide bond. This modification would imply changing both Thr10 and Dab4 for cysteines. In addition, the C-terminal cysteine would need to be derivatized as a carboxamide to mimic the neutral hydroxyethyl threonine moiety being substituted, as shown in Figure 1. Furthermore, Cys10 was introduced with the opposite configuration (d-Cys) to maintain the relative orientation of the moiety.

We also observed in a previous structure–activity study that a norleucine amino acid in position seven yielded more active analogues than the natural leucine in polymyxin. Finally, the branched natural fatty acid tail of polymyxin was substituted by a linear tail.

The minimum inhibitory concentration (MIC) values of several antimicrobials against control and clinical strains are given in Table 1. All clinical isolates were imipenem resistant, and some of them also presented resistance to tobramycin, aztreonam, amikacin, ciprofloxacin, and meropenem.

**Figure 2:**
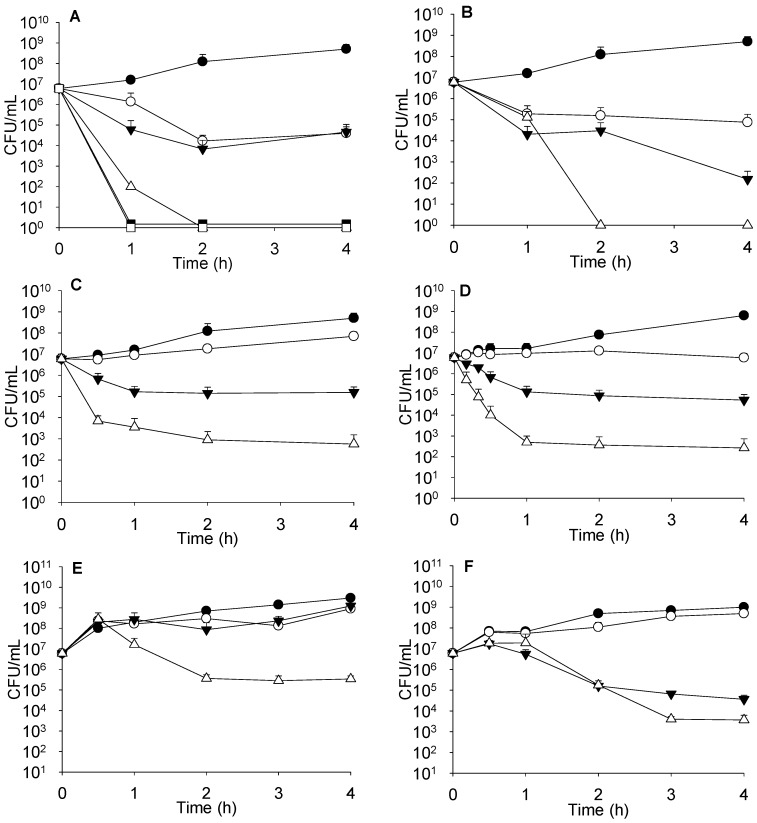
Antibacterial activity of colistin sulphate (COL), imipenem (IMI), meropenem (MER) and AMP38 in different combinations. (**A**) ATCC strain; Control (filled circles), IMI 4 μg/mL (open circles), COL 0.5 μg/mL and IMI 4 μg/mL (filled triangles), COL 4 μg/mL (open triangles), COL 4 μg/mL and IMI 0.5 μg/mL (filled squares), COL 4 μg/mL and IMI 4 μg/mL (open squares); (**B**) PA116136 strain; Control (filled circles), COL 0.25 μg/mL and IMI 32 μg/mL (open circles), COL 2 μg/mL and IMI 4 μg/mL (filled triangles), COL 2 μg/mL and IMI 32 μg/mL (open triangles); (**C**) PA116136 strain; Control (filled circles), IMI 4 μg/mL (open circles), AMP38 8 μg/mL (filled triangles), AMP38 8 μg/mL and IMI 4 μg/mL (open triangles); (**D**) PA116136 strain; Control (filled circles), MER 1 μg/mL (open circles), AMP38 8 μg/mL (filled triangles), AMP38 8 μg/mL and MER 1 μg/mL (open triangles); (**E**) 536SJD strain; Control (filled circles), IMI 4 μg/mL (open circles), AMP38 2 μg/mL (filled triangles), IMI 4 μg/mL and AMP38 2 μg/mL (open triangles); (**F**) 481SJD strain; Control (filled circles), IMI 4 μg/mL (open circles), AMP38 4 μg/mL (filled triangles), IMI 4 μg/mL and AMP38 4 μg/mL (open triangles).

Complementary studies, such as time-kill kinetics or effect on the growth curve have to be performed to accurately evaluate antimicrobial combinations, mostly to investigate the events occurring during the period of first hours. As seen in Figure 2A, combinations of sublethal concentrations of colistin with 4 μg/mL of imipenem did not increase bacterial death. However, when the concentration of colistin was raised to 4 μg/mL and combined with 0.5 or 4 μg/mL of imipenem, bacterial death was markedly enhanced and similar for both imipenem concentrations.

When determining the killing effect on strain PA116136, we found that colistin at 0.25 μg/mL had no effect, but when combined with 4 μg/mL imipenem, a synergistic effect was observed (data not shown). Moreover, higher colistin concentrations (2 μg/mL) exerted a clear concentration-dependent synergistic effect with imipenem, as can be seen in Figure 2B (with 4 and 32 μg/mL imipenem).

Time kill-kinetics with sub-lethal amounts of imipenem and AMP38 are shown in Figure 2C. Assays performed with meropenem are summarized in Figure 2D. Other imipenem-resistant isolates were studied at sublethal concentrations of both AMP38 and imipenem, and with combinations of both (Figure 2E,F). Similar results to those seen in PA116136 were obtained.

**Figure 3:**
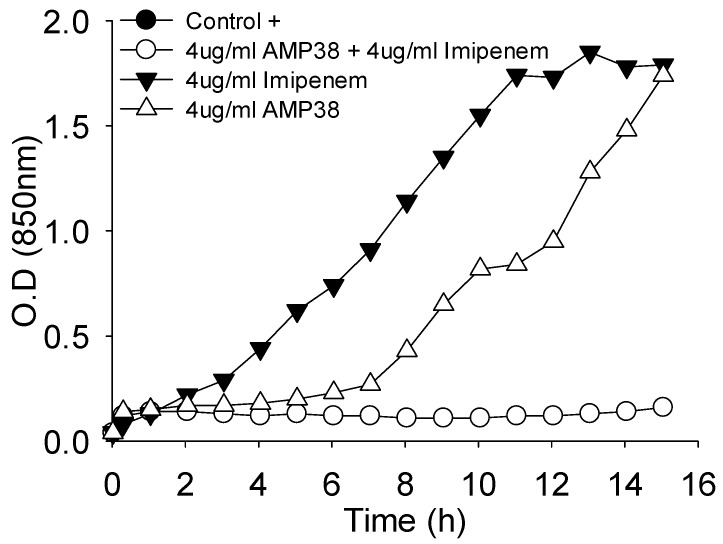
Growth inhibition by the combination of imipenem and AMP38 at 4 μg/mL of each.

Moreover, the combination of both drugs was also assayed for their effect on the growth curve. Figure 3 shows one example where a full inhibition of growth occurred when AMP38 and imipenem were combined at 4 μg/mL of each.

To quantitatively determine the interaction between APMP38 and imipenem, FIC (fractional inhibitory concentration) values were calculated. This index is calculated according to the following formula: FIC of drug A (FIC A) = (MIC of drug A in combination)/(MIC of A); FIC of drug B (FIC B) = (MIC of drug B in combination)/(MIC of B). The FIC index (FICi) is calculated by adding FIC A and FIC B. Table 2 shows FICi values of different bacterial strains when tested with combinations of AMP38 and imipenem.

A biofilm of PA116136 was eradicated by the addition of 62.5 μg/mL of both imipenem and AMP38, whereas imipenem alone failed in eradicating the biofilm at concentrations below 500 μg/mL, and AMP38’s minimal biofilm eradication concentration (MBEC) was higher than 500 μg/mL (Table 3).

**Figure 4:**
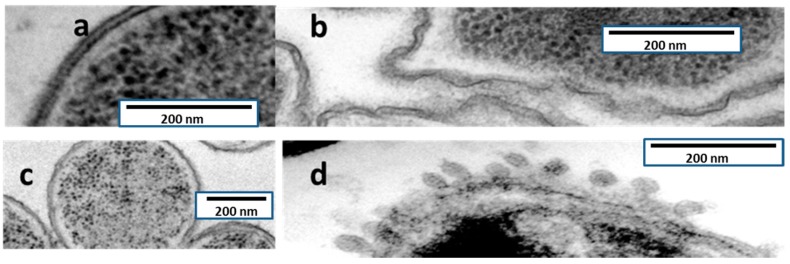
TEM electromicrographs of *P. aeruginosa* PA116136 (**a**) untreated and (**b**) AMP38-treated; and *Serratia marcescens* NIMA strain (**c**) untreated and (**d**) AMP38-treated.

TEM observations of ultrathin sections of both *P. aeruginosa* and *Serratia marcescens* are shown in Figure 4. The effect on the bacterial envelopes is in *S. marcescens* is at least apparently identical to the one produced by colistin (formation of blebs), whereas in *P. aeruginosa*, injuries had a different aspect, lacking blebs and appearing as a rough surface.

Acute toxicity was assessed by an in vivo acute toxicity test on CD-1 mice. The lethal dose (LD50) was determined according to the up-and-down procedure. Compound AMP38 was administered subcutaneously at designated doses (100, 200, and 400 mg/kg). Mice treated with 100 and 200 mg/kg of compound survived with no signs of toxicity; whereas mice administered 400 mg/kg died. After 14 days, necropsy of the surviving mice (dosed at 200 mg/kg) showed no signs of pathology in vital organs. LD50 was determined using the maximum likelihood method, and a value of 283 mg/kg was obtained. This value is significantly higher (almost five times) than the LD50 reported for subcutaneous administration of colistin (59.5 mg/kg). Moreover, preliminary cytotoxicity determination in L-929 and HepG2 cells showed no differences between controls and peptide wells up to 100 μg/mL (data not shown).

## 3. Discussion

Colistin has been reported as synergistic with carbapenems and other antimicrobials in *Acinetobacter baumanii*, as well as in *Pseudomonas* and a few other Gram-negative bacteria [15].

Combinations of colistin and imipenem gave a FICi of 0.625 for ATCC 27853; such a value has to be considered as indifferent. Moreover, when assays were performed with AMP38, the FICi value was 0.625—a value identical to the one determined with colistin and imipenem. This suggests that both imipenem and colistin act in a similar manner on this imipenem-susceptible strain. It should be noted that, in principle, the entry of imipenem in fully susceptible bacteria is not prevented by the outer membrane, since OprD is functional and can allow the penetration of imipenem to the periplasmic space, and subsequently, the antibiotic can reach its target. On the contrary, when acting on imipenem-resistant strains, the combination of imipenem and AMP38 gave FICi values below 0.5; this value has to be considered as strongly synergistic (Table 2). PA116136 is imipenem-resistant, since the OprD gene is knocked out by the presence of an insertion sequence [21]; the rest of the resistant strains are OprD defective. Subsequently, the entry of imipenem is strongly limited in standard culture conditions, and this is the main (if not unique) reason why these bacteria are resistant to imipenem. Thus, the synergism between the peptide and the carbapenem should be interpreted as a consequence of the ability of peptides to open ways by which imipenem can reach PBPs. Similar scenarios were seen in the other resistant strains lacking OprD. Moreover, these results pointed out that the injuries in the outer membrane caused by AMP38 seem to facilitate the entry of imipenem.

FICi values have certain limitations, since they do not give information about the kinetics of bacterial killing. The results are determined after 24 h exposure to antimicrobials, and thus represent a fixed picture of the state after the incubation period. Consequently, with this type of assay, one could disregard the eventual usefulness of antimicrobial combinations for the treatment of complicated microbial infections. Thus, we have performed a series of experiments in order to explore the antimicrobial actions during the first steps of the growth cycle. Combinations of sublethal concentrations of colistin with imipenem failed in increasing bacterial death. On the contrary, the 4 μg/mL concentration of colistin combined with either 0.5 or 4 μg/mL of imipenem markedly enhanced death, irrespective of imipenem concentration; this is consistent with the hypothesis that colistin facilitates the entry of carbapenem, and that eventual resistance would be due to restrictions in the access of imipenem to PBPs (Figure 2A).

Combinations of AMP38 and imipenem enhanced bacterial death. Whereas 8 μg/mL of AMP38 alone had a bacteriostatic effect, the combination of AMP38 (also at 8 μg/mL) plus imipenem (4 μg/mL) resulted in a bactericidal effect—despite the fact that the MIC of imipenem is 16 and that of AMP38 is 32—although the combination failed to completely eradicate the bacterium (Figure 2C). Meropenem at 1 μg/mL had a bacteriostatic effect, keeping bacteria at 10^7^ CFU/mL. Nonetheless, the combination of 4 μg/mL of AMP38 plus 1 μg/mL of meropenem completely killed bacteria after 6 h of contact. 

Time kill-kinetics with sub-lethal amounts of imipenem and AMP38 and assays performed with meropenem (Figure 2D), as well as those at sublethal concentrations of both AMP38 and imipenem and combinations of both (Figure 2E,F) confirmed that AMP38 facilitates the entry of imipenem by disturbing the outer membrane. Figure 3 shows the effect on the mucous 023VH strain growth curve to be very similar to the results obtained with the rest of strains studied (data not shown).

One of the main goals of antimicrobial chemotherapy is the eradication of biofilms. Stable biofilms can play a key role in pathogenesis, and this is particularly a matter of concern in respiratory infections produced by *P. aeruginosa*. This species is able to produce stable biofilms in many situations, particularly when causing lung infections. Sessile bacteria living in a biofilm state are generally more resistant to antimicrobial agents. The ability of combinations of AMP38 and imipenem to eradicate biofilms was explored. In our experience (data not shown,) the use of colistin to eradicate biofilms needs concentrations higher than 1000 μg/mL; imipenem’s MBEC is 500 μg/mL, and that of imipenem is higher than 500 μg/mL, whereas the combination of AMP38 and imipenem was able to completely eradicate the biofilm at 62.5 μg/mL. Thus, a true synergism of imipenem and AMP38 was again observed.

TEM has allowed significant advances in the understanding of bacterial structure and eventually physiology [22], including antibiotic tolerance and biofilm formation. The mechanism of action of AMP38 in *P. aeruginosa* and *S. marcescens* was confirmed by examining untreated and treated bacteria with transmission electron microscopy. Untreated *P. aeruginosa* cells were examined by TEM. Electron micrographs of AMP38-treated *P. aeruginosa* (Figure 4) show a disorganized outer and inner membrane, as compared to the smoothness of untreated membranes. As a control, *S. marcescens* was used. This bacterium is intrinsically resistant to colistin, because although colistin disorganizes its outer membrane, it is not capable of altering the inner membrane [23], and the bacterium remains viable. Typically, the effect of colistin on *S. marcescens* cells is visualized as the production of blebs. We performed an ultrastructural analysis via TEM in order to explore whether colistin and AMP38 share mechanisms of action.

When *S. marcescens* was exposed to colistin, blebs appeared on the outer layer, but no changes were visualized in the cytoplasmic membrane (Figure 4). Figure 4 shows *S. marcescens* exposed to the AMP38, where the same blebs were observed in the outer membrane. Although further research has to be done, the microscopically visualized effects of AMP38 and colistin on Gram-negative bacteria seem to be similar, if not identical.

In conclusion, our data pointed out that the use of synthetic AMPs inspired by natural products that are potentially less toxic [18] than the natural parent compound may contribute to the rescue of antimicrobial agents to which some pathogens have become resistant. In this case, it seems feasible to kill imipenem-resistant *Pseudomonas aeruginosa* with imipenem when combined with other molecules (such as AMP38) which are able to sensitize the bacterium to the antibiotic. Since carbapenems remain the main antimicrobials for treating multidrug-resistant *P. aeruginosa* infections, and the development of carbapenem resistance may significantly compromise their efficacy, the use of peptides such as AMP38 could serve to rescue the use of carbapenems for these purposes. Their use should give better results than the use of colistin and polymyxins, which have already demonstrated synergistic effects [24].

## 4. Experimental Section

### 4.1. Bacterial Strains, Media, and Antimicrobial Substances

The imipenem-resistant *P. aeruginosa* PA116136 isolate was obtained from a patient with chronic pulmonary disease at the Servei de Microbiologia of the Hospital Universitari de Bellvitge (L’Hospitalet de Llobregat, Barcelona, Spain). It has an insertion sequence (ISPa133) sited just before nucleotide 697. This interrupts the coding region, producing a loss of OprD expression and consequently contributes to the resistance to carbapenems [25]. *P. aeruginosa* 536SJD and 481SJD isolates were obtained from patients with chronic pulmonary disease at the Servei de Microbiologia of the Hospital Sant Joan de Déu (Esplugues de Llobregat, Barcelona, Spain). *P. aeruginosa* 023VH and 846VH isolates were obtained from cystic fibrosis patients at the servei de Microbiologia of the Hospital Universitari Vall d’Hebron (Barcelona, Spain). *P. aeruginosa* ATCC 27853 served as a control strain in susceptibility tests. Cation-adjusted Mueller–Hinton (CAMHB, Becton, Dickinson and Company, San Agustin de Guadalix, Madrid, Spain) was used to determine minimum inhibitory concentrations (MICs) and minimal biofilm eradication concentration (MBEC). Tryptic soy agar (TSA, Scharlau Microbiology, Sentmenat, Spain) was used for the determination of colony counts in time-kill assays. *Serratia marcescens* strain nima [26] was used as a control in electron microscopy. Imipenem monohydrate, tobramycin sulfate, amikacin sulfate, ciprofloxacin, and aztreonam were obtained from Sigma-Aldrich (Madrid, Spain); colistin sulphate was kindly supplied from Zhejiang Shenghua Biok Biology Co., Ltd., (Shanghai, China), and the cyclolipopeptide analog of polymyxin AMP38 was synthetized by us.

### 4.2. Peptide Synthesis and Purification

The synthesis of peptide AMP38 was performed manually following standard Fmoc/tBu procedures using DIPCDI/HOBt activation on Rink amide resin. Once the sequence was assembled, cleavage of the peptide from the resin was carried out by acidolysis with Trifluoroacetic acid /triisopropylsilane/water (95:3:2, *v*/*v*) for 90 min. TFA was removed with a stream of nitrogen gas. The oily residue was treated with dry diethyl ether, and the precipitated peptide was isolated by centrifugation. The homogeneity of the crude peptide was assessed by analytical HPLC on Nucleosil C18 reverse-phase columns (4 mm× 250 mm, 5 μm particle diameter, and 120 Å porous size). Elution was carried out at 1 mL·min^−1^ flow with mixtures of H_2_O containing 0.045% TFA and acetonitrile containing 0.036% TFA, with UV detection at 220 nm. Cyclization of the peptide was carried out in 5% dimethylsulphoxide aqueous solution for 24 h and lyophilized twice. The peptide was subsequently purified by preparative HPLC on a Waters DeltaPrep 3000 system with a Phenomenex C18 [18,27,28,29] column (250 mm × 10 mm, 5 μm) eluted with H_2_O/acetonitrile/0.1% TFA gradient mixtures and UV detection at 220 nm. Final purity was greater >99% according to analytical HPLC. The peptide was characterized by matrix-assisted laser desorption ionization time of flight (MALDI-TOF) mass spectrometry with a PerSeptive Biosystems Voyager-DE instrument. MALDI-TOF MS, *m*/*z* (C_55_H_96_N_16_O_12_S_2_): 1237.9 [M + H]^+^, 1259.7 [M + Na]^+^, 1275.7 [M + K]^+^, 1219.9 [M − H_2_O]^+^ (Figure 1).

### 4.3. Determination of Minimum Inhibitory Concentrations

MIC values were determined by broth microdilution method and interpreted according to European Committee on Antimicrobial Susceptibility Testing EUCAST guidelines [30].

### 4.4. Synergy Study

A checkerboard test was used to determine the fractional inhibitory concentrations (FICs) of colistin in combination with the peptide AMP38. Each well in a 96-well plate was inoculated with 100 μL of a bacterial inoculum of 1 × 10^5^ CFU/mL, and the plates were incubated at 37 °C for 24 h. The FIC was calculated after identifying the first well in each row without growth (MIC), according the following formula: FIC of drug A (FIC A) = (MIC of drug A in combination)/(MIC of A); FIC of drug B (FIC B) = (MIC of drug B in combination)/(MIC of B). The FIC index (FICi) values were calculated by adding the FIC of imipenem to the FIC of AMP 38. FICi values were interpreted as follows [21]: FICi < 0.5, synergistic; FICi ≥ 0.5 and < 4, no interaction; FICi > 4, antagonistic [31].

### 4.5. Time–Kill Curves

Killing curve assays were performed with a starting inoculum of 6 × 10^6^ CFU/mL. Strains were tested against colistin, imipenem, meropenem, tobramycin, amikacin, ciprofloxacin, aztreonam, and AMP38 alone and in all possible combinations at concentrations above and below the MICs. Antimicrobials were added to 10 mL of bacteria in the exponential phase of growth and incubated at 37 °C with shaking. Samples were obtained aseptically at 0.5, 1, 2, and 4, diluted in Ringer 1/4 and plated on TSA for colony counting. The response of microbial strains to a single antimicrobial and to the combinations of pairs of antimicrobials was determined by lowering logarithms of viable bacteria.

In accordance with Lora-Tamayo et al. [26], an antimicrobial was considered active when a reduction of ≥1 log10 relative to the initial inoculum was observed. A combination of antimicrobials was considered synergistic when a reduction of ≥2 log10 was observed with respect to the most active antimicrobial. A combination of two antibiotics was considered additive when the logarithm of reduction was between 1 and 2.

### 4.6. Transmission Electron Microscopy

Both *P. aeruginosa* PA116136 and *S. marcescens* nima strains were observed by TEM after treatment with AMP38 or colistin, using untreated bacteria as controls. Bacteria in the exponential phase of growth were centrifuged at 8000× *g* for 10 min to obtain a concentration of 10^8^ CFU/mL. Pellets were then suspended in Tripticase Soy Broth (TSB) and antimicrobials were added (300 mg/L for *S. marcescens* and 100 mg/L for *P. aeruginosa* for both colistin and AMP38). Bacteria were incubated for 20 min at 37 °C and harvested by centrifugation. Bacteria were cryo-immobilized by using a Leica HPM100 high-pressure freezer (Leica, Vienna, Austria). Frozen samples were freeze-substituted (Leica Microsystems, AFS2) for 72 h at −90 °C. Temperature was gradually increased to 4 °C, and then samples were stored at room temperature. Substitutions were performed in pure acetone containing 2% (*w/v*) osmium tetroxide and 0.1% (*w/v*) uranyl acetate. Afterwards, samples were washed with acetone and gradually infiltrated in a resin Epon. Samples were embedded in fresh Epon and polymerized at 60 °C for 48 h. Ultrathin sections (50–70 nm) were made with the Leica Ultracut UC6 (Leica, Vienna, Austria). Epon-embedded thin sections were examined in a JEM1010 JEOL transmission electron microscope (JEOL Ltd., Tokyo, Japan) by working with a tungsten filament at 80 kV. Images were acquired with the software Analysis (version 3.2, Soft Imaging System GmbH, Münster, Germany).

### 4.7. MBEC Determination

Minimal biofilm eradication concentration (MBEC) determinations were conducted as described by Moskowitz et al. [32] with a few modifications. Briefly, bacterial biofilms were formed by immersing the pegs of a modified polystyrene microtiter lid (catalog No. 445497; Nunc TSP system) into 96-well microtiter plates containing 200 μL of CAMHB each, followed by incubation at 37 °C for 24 h in static conditions. Pegs were then gently rinsed in 0.9% NaCl solution, and biofilms were exposed to different concentrations of antimicrobials for 24 h at 37 °C. Pegs were again rinsed with 0.9% NaCl solution and biofilms were removed by 10 min sonication. Recovered bacteria were incubated for 24 h at 37 °C. Optical densities at 620 nm were measured in order to determine MBEC values, defined as the lowest concentration of antimicrobial that prevented bacterial regrowth from the treated biofilm. All experiments were performed in triplicate.

## Figures and Tables

**Table 1:** Antimicrobial susceptibility (minimum inhibitory concentration, MIC) (μg/mL). R: resistant; S: susceptible.

Antimicrobial Agent	ATCC	PA116136	023VH	481SJD	536SJD	846VH
IMIPENEM	4 (S)	16 (R)	16 (R)	32/16 (R)	16 (R)	>32 (R)
COLISTIN	2 (S)	1 (S)	1/2 (S)	1/2 (S)	1/2 (S)	4 (S)
AMP38	4	32	4/2	16	8/16	0.5/1
TOBRAMICIN	1 (S)	1 (S)	16 (R)	2 (S)	2 (S)	2 (S)
AZTREONAM	8 (S)	2 (S)	2 (S)	>32 (R)	>32 (R)	16 (S)
AMIKACIN	0.5 (S)	1 (S)	>32 (R)	4 (S)	2/4 (S)	32 (R)
CIPROFLOXACIN	0.5 (S)	0.25 (S)	0.5 (S)	2/4 (R)	4 (R)	16 (R)
MEROPENEM	0.5 (S)	4 (I)	4 (I)	2 (S)	4/8 (I)	32 (R)

**Table 2:** Fractional inhibitory concentration index (FICi) values of imipenem and AMP38 combination.

STRAINS	FICi
ATCC	0.62
PA116136	0.14
023VH	0.18
481SJD	0.12
536SJD	0.07

**Table 3:** Minimal biofilm eradication concentration (MBEC) of imipenem, AMP38, and the combination of both antimicrobials (μg/mL).

Antimicrobial Agent	PA116136
IMIPENEM	>500
AMP38	500
IMIPENEM + AMP38	62.5
